# Alcohol consumption and breast tumor gene expression

**DOI:** 10.1186/s13058-017-0901-y

**Published:** 2017-09-12

**Authors:** Jun Wang, Yujing J. Heng, A. Heather Eliassen, Rulla M. Tamimi, Aditi Hazra, Vincent J. Carey, Christine B. Ambrosone, Victor P. de Andrade, Adam Brufsky, Fergus J. Couch, Tari A. King, Francesmary Modugno, Celine M. Vachon, David J. Hunter, Andrew H. Beck, Susan E. Hankinson

**Affiliations:** 1 Department of Biostatistics and Epidemiology, School of Public Health and Health Sciences, University of Massachusetts Amherst, 715 N Pleasant Street, Amherst, MA 01003 USA; 2 Present address: Department of Preventive Medicine, University of Southern California, Harlyne J. Norris Research Tower, 1450 Biggy Street, Los Angeles, CA 90033 USA; 3 Department of Pathology, Beth Israel Deaconess Medical Center and Harvard Medical School, Boston, MA 02215 USA; 4 Channing Division of Network Medicine, Department of Medicine, Brigham and Women’s Hospital and Harvard Medical School, 181 Longwood Avenue, Boston, MA 02115 USA; 5 Department of Epidemiology, Harvard T.H. Chan School of Public Health, 677 Huntington Avenue, Boston, MA 02115 USA; 6 Division of Preventive Medicine, Department of Medicine, Brigham and Women’s Hospital and Harvard Medical School, 900 Commonwealth Ave, Boston, MA 02115 USA; 7 Department of Cancer Prevention and Control, Roswell Park Cancer Institute, Elm & Carlton Streets, Buffalo, NY 14263 USA; 8 Departamento de Patologia, A.C. Camargo Cancer Center, São Paulo, SP 01509-900 Brazil; 9 Department of Medicine, University of Pittsburgh Medical Center, 300 Halket Street, Pittsburgh, PA 15213 USA; 10 Department of Laboratory Medicine and Pathology, Mayo Clinic, 200 First Street SW, Rochester, MN 55905 USA; 11 Dana-Farber Cancer Institute and Brigham and Women’s Cancer Center, 450 Brookline Avenue, Boston, MA 02215 USA; 12 Department of Obstetrics, Gynecology and Reproductive Sciences, University of Pittsburgh School of Medicine, 300 Halket Street, Pittsburgh, PA 15213 USA; 13 Department of Health Sciences Research, Mayo Clinic, 200 First Street SW, Rochester, MN 55905 USA; 14 Department of Nutrition, Harvard T.H. Chan School of Public Health, 677 Huntington Avenue, Boston, MA 02115 USA

**Keywords:** Alcohol, Prospective, Epidemiology, Breast tumor, Gene expression

## Abstract

**Background:**

Alcohol consumption is an established risk factor for breast cancer and the association generally appears stronger among estrogen receptor (ER)-positive tumors. However, the biological mechanisms underlying this association are not completely understood.

**Methods:**

We analyzed messenger RNA (mRNA) microarray data from both invasive breast tumors (*N* = 602) and tumor-adjacent normal tissues (*N* = 508) from participants diagnosed with breast cancer in the Nurses’ Health Study (NHS) and NHSII. Multivariable linear regression, controlling for other known breast cancer risk factors, was used to identify differentially expressed genes by pre-diagnostic alcohol intake. For pathway analysis, we performed gene set enrichment analysis (GSEA). Differentially expressed genes or enriched pathway-defined gene sets with false discovery rate (FDR) <0.1 identified in tumors were validated in RNA sequencing data of invasive breast tumors (*N* = 166) from The Cancer Genome Atlas.

**Results:**

No individual genes were significantly differentially expressed by alcohol consumption in the NHS/NHSII. However, GSEA identified 33 and 68 pathway-defined gene sets at FDR <0.1 among 471 ER+ and 127 ER- tumors, respectively, all of which were validated. Among ER+ tumors, consuming 10+ grams of alcohol per day (vs. 0) was associated with upregulation in RNA metabolism and transport, cell cycle regulation, and DNA repair, and downregulation in lipid metabolism. Among ER- tumors, in addition to upregulation in RNA processing and cell cycle, alcohol intake was linked to overexpression of genes involved in cytokine signaling, including interferon and transforming growth factor (TGF)-β signaling pathways, and translation and post-translational modifications. Lower lipid metabolism was observed in both ER+ tumors and ER+ tumor-adjacent normal samples. Most of the significantly enriched gene sets identified in ER- tumors showed a similar enrichment pattern among ER- tumor-adjacent normal tissues.

**Conclusions:**

Our data suggest that moderate alcohol consumption (i.e. 10+ grams/day, equivalent to one or more drinks/day) is associated with several specific and reproducible biological processes and pathways, which adds potential new insight into alcohol-related breast carcinogenesis.

**Electronic supplementary material:**

The online version of this article (doi:10.1186/s13058-017-0901-y) contains supplementary material, which is available to authorized users.

## Background

Alcohol consumption is an established breast cancer risk factor [1]. Large prospective cohort studies have reported a modest but significant increase in risk (8–9%) per 10 g of alcohol consumed per day [1, 2]. Specifically, in the Nurses’ Health Study (NHS) with long-term average alcohol consumption, the risk increased by 15% (95% confidence interval (CI) 1.06–1.24) for 5.0–9.9 g/day of alcohol and by 51% (95% CI 1.35–1.70) for at least 30 g/day of alcohol, compared to women who did not drink [3]. The positive association was observed in both estrogen receptor (ER)-positive (ER+) and ER-negative (ER-) tumors but appeared stronger with ER+ than with ER- tumors [3, 4].

The mechanism underlying the alcohol and breast cancer association is not completely understood. One major hypothesis is that this association is mediated, at least in part, through estrogen metabolism [5, 6]. Other hypothesized mechanisms include the generation of acetaldehyde and reactive oxygen species (ROS) during alcohol metabolism [7]. Acetaldehyde has been classified as a carcinogen by the International Agency for Research on Cancer (IARC) [8] and, after alcohol administration, accumulation of acetaldehyde was observed in rat mammary tissue in experimental studies [9, 10]. Ethanol oxidation can lead to generation of ROS in rat mammary tissue [9, 10] and ROS promotes many aspects of tumor development and progression [11]. In addition, disruption of folate metabolism and DNA and/or histone hypomethylation have been hypothesized to be involved in alcohol-mediated carcinogenesis [8]. However, despite these hypotheses, no definitive mechanisms have yet been identified.

Assessment of molecular and/or genetic markers in breast tumor tissues may provide insights into the underlying mechanism(s) for established breast cancer risk factors. Recent studies evaluating breast tumor genome-wide gene expression profiling have identified molecular signatures associated with several established risk factors, such as body mass index (BMI) [12] and parity [13]. However, to date, no studies have assessed alcohol-related molecular signatures in breast tumors. To help unravel the underlying mechanisms of alcohol consumption and breast cancer risk, we evaluated the association between pre-diagnostic alcohol consumption and genome-wide gene expression in breast tumor and tumor-adjacent normal tissue in the prospective NHS and NHSII, and further validated our results in an independent validation dataset obtained from The Cancer Genome Atlas (TCGA) [14]. We hypothesized that the biological pathways underlying the association between alcohol and breast cancer could vary by tumor ER status and thus conducted the analysis by tumor ER expression.

## Methods

### Study population

The NHS was established in 1976 when 121,700 US female registered nurses, aged 30–55 years, completed an initial mailed questionnaire, and the NHSII was established in 1989, when 116,429 US female registered nurses, aged 25–42 years, completed and returned an initial questionnaire. Both cohorts have been followed biennially by mailed questionnaire to update information on exposure status and ascertain newly diagnosed diseases, including cancers. All women reporting incident diagnoses of breast cancer were asked for permission to review their medical records; cases for which pathology reports were obtained were confirmed by medical record review (>99%).

For this analysis, we included invasive breast cancer cases with both sufficient RNA from formalin-fixed paraffin-embedded (FFPE) tumor blocks for expression profiling and with available blood samples (the latter criterion to maximize the utility of the subset of cases that could be arrayed). Upon meeting the two criteria, in the NHS, we identified 532 invasive postmenopausal cases diagnosed in 1990–2004 which were a subset of the Cancer Genetic Markers of Susceptibility (CGEMS) initiative [15]; in the NHSII, invasive cases, regardless of menopausal status, diagnosed in 1995–2009 in the NHSII (N = 280) were included. Archived FFPE breast tumor blocks were obtained from the cohort tumor tissue repository; details of breast tumor tissue block collection have been described previously [16, 17]. Although only a subset of all the eligible cases were included in the TMA (primarily because either the tumor blocks had been destroyed by the hospital or there was insufficient tumor in the block), in each cohort, the characteristics of participants included in the TMA were very similar to those of all the eligible cases, including alcohol consumption and other breast cancer risk factors (e.g. first-degree family history, BMI and parity). The study was approved by the Committee on the Use of Human Subjects in Research at the Brigham and Women’s Hospital.

### Assessment of alcohol exposure and other covariates

The assessment of alcohol consumption has been reported in detail elsewhere [3]. Briefly, information was first collected in 1980 in the NHS and in 1991 in the NHSII when participants reported their average frequency of intake for each alcoholic beverage (i.e. beer, wine, and liquor) during the previous 12 months through a semi-quantitative food frequency questionnaire, which was updated every 2–4 years thereafter in each cohort. Alcohol intake (grams per day) was calculated as the sum of the daily number of drinks multiplied by the average alcohol content of each beverage type (12.8 g per beer, 11.0 g per glass of wine, and 14.0 g per serving of liquor). We then calculated cumulative average intake by averaging alcohol consumption over time using all available information beginning in 1980 (NHS) or 1991 (NHSII). We also evaluated recent alcohol intake using information from the questionnaire cycle before diagnosis (i.e. 2–4 years before diagnosis). Cumulative average and recent alcohol consumption were highly correlated (Spearman *r* = 0.87) and results were very similar when using either cumulative average or recent alcohol; thus we presented results from recent alcohol intake. Covariate data, including parity, family history of breast cancer, BMI (weight(kg)/height(m)^2^), menopausal status and menopausal hormone therapy (MHT) use were obtained from the NHS or NHSII questionnaire at baseline and subsequent biennial questionnaires; for BMI, menopausal status and MHT use, the information taken from the most recent questionnaire was used.

### Gene expression microarray and quality control analysis

RNA was extracted from multiple cores of 1 or 1.5 mm taken from tumor (*N* = 3 cores) or adjacent normal (*N* = 5 cores) tissues from FFPE blocks using the Qiagen AllPrep RNA isolation kit. Tumor-adjacent normal tissue was generally > 1 cm from the tumor edge. Since FFPE samples are known to have variable yields, tissues from all the cores from the same patient were placed into one microtube to maximize RNA yield. Total RNA was used to synthesize double-stranded complementary DNA which was then fragmented and hybridized to Affymetrix Glue Grant Human Transcriptome Array [18] (HTA 3.0v1 pre-release version from Affymetrix, Santa Clara, CA, USA). We included four independent breast tumor samples as technical replicates (identified from Beth Israel Deaconess Medical Center, Boston, MA, USA) in each assay plate; the correlation of these replicates across all arrays was ≥ 0.93.

Gene expression data were normalized and summarized using robust multiarray average (RMA; Affymetrix Power Tools (APT) v1.18.0). Out of the total 1324 tumor and tumor-adjacent normal specimens (934 and 390 in the NHS and NHSII, respectively), we excluded 131 (14%) and 43 (11%) from the NHS and NHSII, respectively, with an area under the curve (AUC) < 0.55 (evaluated using APT probeset summarization-based metrics) and further excluded 40 that failed the non-outlier analysis by arrayQualityMetrics v3.24.0 [19], leaving 1110 samples (602 tumors and 508 tumor-adjacent normal samples) for analysis. Although tumor specimens from the NHS were generally older than those from the NHSII, the proportions filtered out according to RNA quality (i.e. 14% vs. 11%) were similar in the two cohorts. Non-specific filtering by median expression levels was used to remove the bottom 25% of expressed probes, leaving 25,979 gene-level annotated transcript clusters included in the analysis. Gene expression data were deposited into the Gene Expression Omnibus [GEO: GSE93601].

We also assessed biological concordance (i.e. probe expression concordance with protein markers measured by immunohistochemical (IHC) staining) for select probes. We confirmed the correlation between probes for ESR1, PGR, and ERBB2 with IHC markers, ER, progesterone receptor (PR), and human epidermal growth factor receptor 2 (HER2), in tumors to confirm biological reproducibility of the data (Additional file 1: Figure S1).

### Statistical analysis

**Fig. 1:**
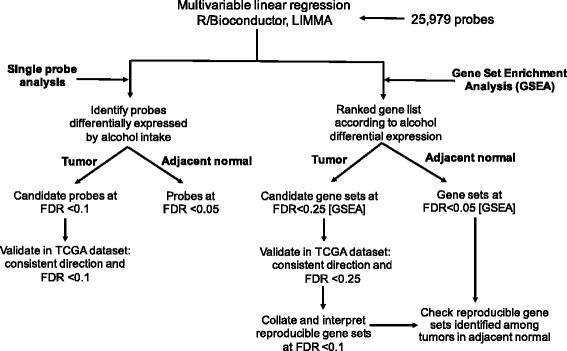
Analysis strategy for identifying differentially expressed probes or enriched pathway-defined gene sets in the Nurses’ Health Study (NHS) and the NHSII. FDR false discovery rate, LIMMA linear models for microarray data

We performed analyses at the level of both probes and pathway-defined gene sets (Fig. 1). All analyses were conducted separately in ER+ tumors, ER+ tumor-adjacent normal tissues, and ER- tumors and ER- tumor-adjacent normal tissues. We conducted multivariable linear regression using the R Bioconductor package linear models for microarray data (LIMMA) [20] for 25,979 probes. To maximize power, samples from the NHS and NHSII were pooled in all analyses (although NHS and NHSII samples were run on different plates, all samples were normalized together) and we adjusted for microarray plate, thus controlling for both cohort and plate, in the regression models. Alcohol consumption was defined as a three-category variable: 0, > 0 to < 10 and 10+ g/day. Factors correlated with alcohol consumption and/or those known to affect tumor gene expression were evaluated as potential covariates in the regression models. Age at diagnosis, year of diagnosis, microarray plate, first-degree family history of breast cancer and recent BMI were included in the final models presented here. Although smoking status is often correlated with alcohol intake, smoking was not adjusted for in the analysis because so few women (~8%) were current smokers 2–4 years before diagnosis. In the single-probe analysis (*N* = 25,979 probes), individual probes were considered significantly differentially expressed by alcohol intake using a false discovery rate (FDR) threshold: FDR <0.1 for tumors and FDR <0.05 for tumor-adjacent normal tissues (due to the lack of a validation dataset of tumor-adjacent normal samples, a more stringent FDR threshold was applied).

To incorporate biological knowledge into the analysis, we further performed gene set enrichment analysis (GSEA) [21] to identify pathway-defined gene sets that varied by alcohol intake. Gene sets were collected from the Molecular Signatures Database (MSigDB) (http://www.broadinstitute.org/gsea/msigdb/), including 217 from BioCarta, 186 from Kyoto Encyclopedia of Genes and Genomes (KEGG), 674 from Reactome, and 825 from Gene Ontology (GO) biological process; those with < 15 genes or > 500 genes were filtered out, leaving 1293 pathway-defined gene sets in the analysis. The four pathway databases were included because each has a distinct but also complementary approach to capture known biological pathways [22]. We used the GSEA “Pre-ranked” function and imported ranked gene lists according to the alcohol-associated *t* statistic from the regression models. In the pathway analysis, only probes that are annotated as a gene (*N* = 15,407 probes) were included in the GSEA. Briefly, all the genes were first ranked according to the alcohol-associated *t* statistic; an enrichment score was then calculated for each gene set. The enrichment score corresponds to a weighted Kolmogorov-Smirnov-like statistic and reflects the extent to which the gene set is overrepresented at the extreme (i.e. top or bottom) of the entire ranked list [21]. If the enrichment score is positive (e.g. the gene set is overrepresented by top ranked genes), then the gene set is considered upregulated while it is considered downregulated if the score is negative. In the discovery stage, among tumors, gene sets at FDR <0.25 were considered significantly enriched. Again, a more stringent FDR threshold (i.e. FDR <0.05) was applied to tumor-adjacent normal samples. We further performed leading-edge subset analysis to identify the core set (i.e. key genes) of the gene set that accounted for the enrichment signal [21].

### Validation analysis

The validation dataset consisted of RNA sequencing (RNA-Seq) data from 166 invasive breast tumors, a subset of breast tumor samples from TCGA that had pre-diagnostic alcohol consumption (generally defined as recent intake) and comparable covariate data. For the validation dataset, we originally contacted six TCGA sites with the largest number of potential cases and four of them agreed to collect or provide already available breast cancer risk factor data, including the University of Pittsburgh, Roswell Park Cancer Institute, the Mayo Clinic and Memorial Sloan Kettering Cancer Center. A total of 220 invasive cases had RNA-Seq data and at least some of the key covariates (e.g. BMI or alcohol or parity), of which 166 had complete information on alcohol consumption and covariates that were required for adjustment in the regression models. TCGA RNA-Seq data were previously processed using the MapSplice algorithm [23] to perform the alignment and RNA-Seq by expectation maximization (RSEM) [24] to estimate gene abundance. The expression dataset included 20,531 genes; in the differential expression analysis, genes with low expression (i.e. < 25^th^ percentile) according to median counts per million were removed, leaving 15,398 unique genes. The common genes in the NHS/NHSII and the TCGA dataset accounted for approximately 84% of all the genes in each dataset. The RNA-Seq data were normalized using the trimmed mean of M-values [25] and log-transformed with associated precision weights using Voom. Multivariable linear regression implemented through R/Bioconductor LIMMA was then used to identify genes that were differentially expressed by recent alcohol intake and we further performed GSEA using similar methods as in the NHS/NHSII.

To validate the significantly enriched pathway-defined gene sets identified in the NHS/NHSII, we required that these gene sets showed a consistent direction (i.e. same upregulation or downregulation) of enrichment and an FDR <0.25 in the TCGA dataset (Fig. 1). Among those replicated gene sets, we only reported gene sets at FDR <0.1 in the NHS/NHSII. No validation dataset of breast normal or tumor-adjacent normal samples with alcohol consumption information was available and thus it was not feasible for us to replicate our results in further datasets.

## Results

In the NHS and NHSII, the average alcohol intake was relatively low (mean 6.4 g/day, SD 11.4). Approximately 34% of the women had no recent alcohol consumption and 45% women consumed < 10 g of alcohol per day and only 21% women consumed 10+ g/day of alcohol. Age at diagnosis and parity were roughly evenly distributed across categories of recent alcohol intake. Women with higher alcohol intake were less likely to have a first-degree family history of breast cancer, had lower BMI and were diagnosed in more recent years (Table 1). Among women with natural menopause or bilateral oophorectomy, those with alcohol intake at least 10 g/day were less likely to use MHT compared to women with no or lower alcohol intake. Out of the 602 tumor specimens, 445 (74%) had matched adjacent normal tissues. The characteristics of women with tumor-adjacent normal samples were similar to those with only tumor specimens available (data not shown). Alcohol consumption and other risk factors such as age at diagnosis and BMI were similar between women diagnosed with ER+ tumors and those with ER- tumors (Table 2). Compared to ER+ tumors, ER- tumors tended to be larger, moderately or poorly differentiated, and diagnosed at a later stage.

**Table 1:** Characteristics of patients with invasive breast cancer according to recent alcohol consumption in the NHS and the NHSII

	Recent alcohol consumption, g/day					
	0		> 0 to < 10		10+	
	*N* = 206		*N* = 267		*N* = 126	
	Mean	SD	Mean	SD	Mean	SD
Age at diagnosis, years	61.6	9.6	59.9	9.0	62.3	9.8
BMI at diagnosis, kg/m^2^	27.3	5.7	25.7	4.6	24.5	4.3
Parity	2.6	1.7	2.7	1.7	2.5	1.9
Cumulative average alcohol^a^, g/day	0.8	2.1	4.2	4.0	19.4	12.0
Recent alcohol, g/day	0	0	3.7	2.6	22.9	16.0
	N	%	N	%	N	%
First-degree family history	38	18.4	45	16.9	13	10.3
Menopausal at diagnosis						
Premenopausal	32	15.5	56	21.0	15	11.9
Postmenopausal	169	82.0	203	76.0	108	85.7
Unknown	5	2.4	8	3.0	3	2.4
Current MHT use^b^	86	52.4	117	60.0	46	43.8
Year of diagnosis						
1990‒1999	122	59.2	157	58.8	66	52.4
2000‒2004	66	32.0	90	33.7	52	41.3
2005‒2009	18	8.7	20	7.5	8	6.3

*NHS* Nurses’ Health Study, *BMI* body mass index, *MHT* menopausal hormone therapy

^a^Calculated as average alcohol consumption over time prior to breast cancer diagnosis using all available exposure information

^b^Current MHT use among postmenopausal women only

**Table 2:** Study population and tumor characteristics by ER status in the NHS and the NHSII

	ER+ tumors *N* = 471		ER- tumors *N* = 127	
	Mean	SD	Mean	SD
Age at diagnosis, years	61.4	9.6	59.1	8.9
BMI at diagnosis, kg/m^2^	26.0	5.0	26.1	5.1
Parity	2.6	1.8	2.9	1.8
Recent alcohol, g/day	6.5	11.7	5.8	10.0
Cumulative average alcohol, g/day	6.4	9.5	5.5	8.2
	N	%	N	%
First-degree family history	79	16.8	16	12.6
Year of diagnosis				
1990‒1999	251	53.3	91	71.7
2000 − 2004	182	38.6	26	20.5
2005‒2009	38	8.1	10	7.9
Tumor size				
0.1‒2.0 cm	365	77.5	68	53.5
2.1‒4.0 cm	75	15.9	46	36.2
> 4.0 cm	23	4.9	7	5.5
Unknown	8	1.7	6	4.7
Lymph node involvement				
None	349	74.1	95	74.8
1‒3 positive nodes	85	18.0	22	17.3
> 3 positive nodes	33	7.0	8	6.3
Metastatic at diagnosis	4	0.8	1	0.8
Unknown	0	0	1	0.8
Grade				
Well-differentiated	124	26.3	6	4.7
Moderately differentiated	243	51.6	35	27.6
Poorly differentiated	89	18.9	67	52.8
Unknown	15	3.2	19	15.0
Stage^a^				
I	305	64.8	57	44.9
II	122	25.9	59	46.5
III	40	8.5	9	7.1
IV	4	0.8	1	0.8
Unknown	0	0	1	0.8

*ER* estrogen receptor, *NHS* Nurses’ Health Study, *BMI* body mass index

^a^Staging was based on tumor size and lymph node involvement

Patients with invasive breast cancer in the validation dataset (i.e. TCGA) were younger at diagnosis, had a higher BMI, were less likely to drink alcohol and were diagnosed more recently (i.e. 2005–2009), compared to those in the NHS/NHSII (Additional file 2: Table S1). In addition, the TCGA dataset included a greater percentage of premenopausal women than the NHS/NHSII dataset (38% vs. 17%); among postmenopausal women, those in the TCGA dataset were less likely to use MHT than women in the NHS/NHSII dataset. While the majority of the TCGA tumors were stage II or III, about 60% of the tumors in the NHS/NHSII were stage I. Similar to the NHS/NHSII, in TCGA, women with higher alcohol intake (i.e. 1+ drink per day) had a lower BMI, were less likely to have a first-degree family history of breast cancer, and tended to be premenopausal/perimenopausal and were diagnosed in more recent years (i.e. 2008–2011), compared to women with lower alcohol intake (Additional file 2: Table S2).

In the single-probe analysis, after adjusting for multiple comparisons, no probes were significantly differentially expressed by recent alcohol consumption (i.e. 10+ vs. 0 g/day) in tumor or tumor-adjacent normal samples (Additional file 2: Table S3 and Additional file 3: Figure S2). When comparing alcohol intake < 10 vs. 0 g/day, two probes showed significantly decreased expression in ER- tumors only (FDR = 0.05, Additional file 2: Table S3); however, no such significantly decreased expression was observed when comparing alcohol intake of 10+ vs. 0 g/day.

**Fig. 2:**
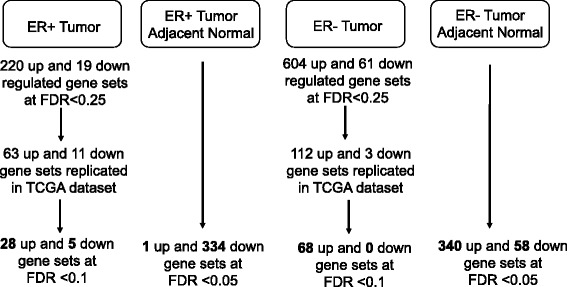
Number of enriched pathway-defined gene sets by alcohol (10+ vs. 0 g/day) in the Nurses’ Health Study (NHS) and the NHSII. ER estrogen receptor, FDR false discovery rate, TGCA The Cancer Genome Atlas

**Fig. 3:**
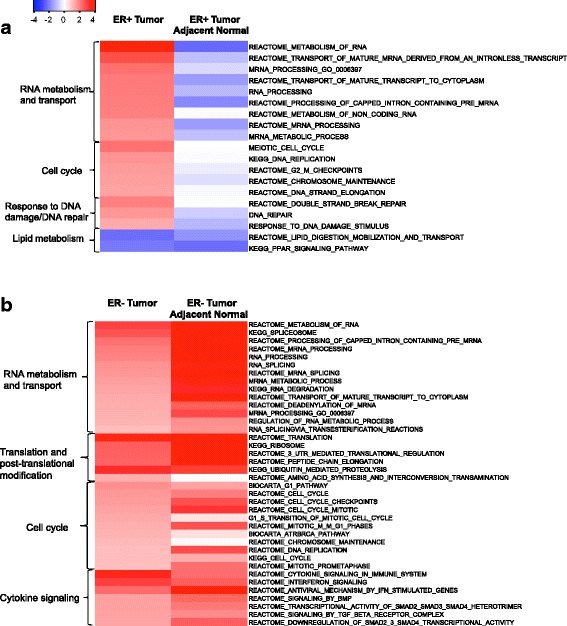
**a**, **b** Replicated enriched pathway-defined gene sets (false discovery rate (FDR) <0.1) by alcohol consumption in the Nurses’ Health Study (NHS) and the NHSII. Replicated significantly enriched gene sets according to recent alcohol intake (i.e. 10+ vs. 0 g/day) in estrogen receptor (ER)+ tumors and the same gene sets in tumor-adjacent normal tissues (**a**), and in ER- tumors and the same gene sets in tumor-adjacent normal tissues (**b**), NHSI/II. The primary biological processes observed are shown: *N* = 19 and 38 gene sets for ER+ and ER- tumors, respectively; -log10(FDR)*direction: red indicates upregulation and blue downregulation

In contrast to the single-probe analysis, we observed significant enrichment for 239 pathway-defined gene sets (FDR <0.25) among ER+ tumors when comparing recent alcohol intake of 10+ g/day with 0 g/day, including 220 upregulated and 19 downregulated gene sets (Fig. 2). Out of the 220 upregulated gene sets, 63 (28.6%) were replicated in TCGA, of which 28 were at FDR <0.1 (Table 3 and Additional file 2: Table S4); out of the 19 downregulated gene sets, 11(57.9%) were replicated, of which 5 were at FDR <0.1 (Table 3). Among the replicated and significantly enriched (FDR <0.1) gene sets in ER+ tumors, alcohol intake (i.e. 10+ vs. 0 g/day) was associated with overexpression of genes involved in RNA metabolism and transport (e.g. REACTOME_METABOLISM_OF_RNA), cell cycle (e.g. GO_MEIOTIC_CELL_CYCLE), DNA repair (e.g. REACTOME_DOUBLE_STRAND_BREAK_REPAIR), downregulation of lipid metabolism (i.e. REACTOME_LIPID_DIGESTION_MOBILIZATION_AND_TRANSPORT) and PPAR signaling pathway (i.e. KEGG_PPAR_SIGNALING_PATHWAY). As there were multiple pathway-defined gene sets linking to similar biological processes, we noted that these gene sets contained both common and distinct genes. For instance, the leading-edge subset analysis revealed that among the three DNA repair related gene sets, there were six common genes (i.e. *ATM*, *LIG1*, *NBN*, *RAD50*, *RAD52* and *RPA1*) which accounted for 12%, 43% and 10% of the key genes (i.e. leading-edge subsets) in the gene set GO_DNA_REPAIR, REACTOME_DOUBLE_STRAND_BREAK_REPAIR and GO_RESPONSE_TO_DNA_DAMAGE_STIMULUS, respectively. In contrast to ER+ tumors, among ER+ tumor-adjacent normal specimens, there was no enrichment for cell cycle related gene sets, and several gene sets of RNA processing were significantly enriched but downregulated (Fig. 3a). However, in both ER+ tumors and tumor-adjacent normal, alcohol consumption was associated with significant downregulation in lipid metabolism and in the PPAR signaling pathway. The PPAR signaling pathway consists of three subfamilies (i.e. alpha, gamma and delta) and the leading-edge subset analysis among ER+ tumors revealed that PPARG specifically was among the core genes that accounted for the enrichment signal.

**Table 3:** Enriched gene sets^a^ by recent alcohol consumption^b^ in ER+ tumors in the NHS and the NHSII

Pathway-defined gene set	Number of enriched genes	NES	FDR
Upregulated			
REACTOME_NONSENSE_MEDIATED_DECAY_ENHANCED_BY_THE_EXON_JUNCTION_COMPLEX	106	2.56	<0.0001
REACTOME_INFLUENZA_LIFE_CYCLE	134	2.49	<0.0001
REACTOME_METABOLISM_OF_RNA	248	2.14	0.001
REACTOME_NEP_NS2_INTERACTS_WITH_THE_CELLULAR_EXPORT_MACHINERY	26	1.99	0.007
REACTOME_TRANSPORT_OF_MATURE_MRNA_DERIVED_FROM_AN_INTRONLESS_TRANSCRIPT	31	1.98	0.008
REACTOME_TRANSPORT_OF_RIBONUCLEOPROTEINS_INTO_THE_HOST_NUCLEUS	26	1.97	0.008
REACTOME_ANTIVIRAL_MECHANISM_BY_IFN_STIMULATED_GENES	61	1.93	0.014
POSITIVE_REGULATION_OF_TRANSCRIPTION_FROM_RNA_POLYMERASE_II_PROMOTER	61	1.93	0.015
REACTOME_INTERACTIONS_OF_VPR_WITH_HOST_CELLULAR_PROTEINS	31	1.90	0.018
MRNA_PROCESSING_GO_0006397	67	1.89	0.019
REACTOME_SYNTHESIS_OF_GLYCOSYLPHOSPHATIDYLINOSITOL_GPI	17	1.89	0.019
MEIOTIC_CELL_CYCLE	24	1.89	0.020
REACTOME_TRANSPORT_OF_MATURE_TRANSCRIPT_TO_CYTOPLASM	51	1.86	0.025
RNA_PROCESSING	159	1.85	0.027
REACTOME_PROCESSING_OF_CAPPED_INTRON_CONTAINING_PRE_MRNA	132	1.83	0.028
REACTOME_METABOLISM_OF_NON_CODING_RNA	45	1.83	0.029
REACTOME_DOUBLE_STRAND_BREAK_REPAIR	18	1.84	0.030
PROTEIN_RNA_COMPLEX_ASSEMBLY	60	1.84	0.030
REACTOME_REGULATION_OF_GLUCOKINASE_BY_GLUCOKINASE_REGULATORY_PROTEIN	26	1.78	0.048
Downregulated			
REACTOME_LIPID_DIGESTION_MOBILIZATION_AND_TRANSPORT	38	−2.12	0.017
REACTOME_SMOOTH_MUSCLE_CONTRACTION	21	−2.13	0.024
REACTOME_MUSCLE_CONTRACTION	40	−2.06	0.025
KEGG_PPAR_SIGNALING_PATHWAY	58	−2.03	0.025

*ER* estrogen receptor, *NHS* Nurses’ Health Study, *NES* normalized enrichment score, *FDR* false discovery rate

^a^Only gene sets replicated in The Cancer Genome Atlas (TCGA) dataset and FDR <0.05 are shown

^b^Enriched gene sets for comparison of recent alcohol consumption 10+ vs. 0 g/day

For ER- tumors, we observed significant enrichment for 665 pathway-defined gene sets (FDR <0.25) when comparing recent alcohol intake of 10+ g/day with 0 g/day, including 604 upregulated and 61 downregulated gene sets (Fig. 2). Out of the 604 upregulated gene sets, 112 (18.5%) were replicated in TCGA, of which 68 were at FDR <0.1 (Table 4 and Additional file 2: Table S5); out of the 61 downregulated gene sets, 3 (4.9%) were replicated but none were at FDR <0.1. The 68 reproducible and significantly (FDR <0.1) upregulated gene sets identified among ER- tumors demonstrated that, in addition to the upregulation in RNA processing and cell cycle regulation, alcohol intake was also linked to strong enrichment in cytokine signaling (e.g. REACTOME_INTERFERON_SIGNALING and REACTOME_SIGNALING_BY_TGF_BETA_RECEPTOR_COMPLEX) and translation and post-translational modification (e.g. KEGG_UBIQUITIN_MEDIATED_PROTEOLYSIS). Among the cytokine signaling pathways, four gene sets were related to TGF-β/SMAD/BMP signaling; four common genes (i.e., *SMAD4*, *SMURF2*, *UBE2D3*, and *UBE2D1*) were observed among the leading-edge subsets of these four gene sets, and the overlapping genes accounted for about 15%, 50%, 21% and 29% of the leading-edge subset of REACTOME_SIGNALING_BY_TGF_BETA_RECEPTOR_COMPLEX, REACTOME_DOWNREGULATION_OF_SMAD2_3_SMAD4_TRANSCRIPTIONAL_ACTIVITY, and REACTOME_TRANSCRIPTIONAL_ACTIVITY_OF_SMAD2_SMAD3_SMAD4_HETEROTRIMER, and REACTOME_SIGNALING_BY_BMP, respectively. Similar significant enrichment was also observed among ER- tumor-adjacent normal tissues (Fig. 3b).

**Table 4:** Enriched gene sets^a^ by recent alcohol consumption^b^ in ER- tumors in the NHS and the NHSII

Pathway-defined gene set	Number of enriched genes	NES	FDR
Upregulated			
REACTOME_TRANSLATION	144	2.31	0.0002
REACTOME_CYTOKINE_SIGNALING_IN_IMMUNE_SYSTEM	218	2.32	0.0003
KEGG_UBIQUITIN_MEDIATED_PROTEOLYSIS	124	2.38	0.001
REACTOME_METABOLISM_OF_MRNA	206	2.25	0.001
PROTEIN_RNA_COMPLEX_ASSEMBLY	60	2.25	0.001
REACTOME_INTERFERON_SIGNALING	129	2.21	0.001
REACTOME_METABOLISM_OF_RNA	248	2.18	0.001
REACTOME_SRP_DEPENDENT_COTRANSLATIONAL_PROTEIN_TARGETING_TO_MEMBRANE	108	2.17	0.001
REACTOME_INFLUENZA_LIFE_CYCLE	134	2.13	0.002
KEGG_SPLICEOSOME	122	2.10	0.002
KEGG_RIBOSOME	84	2.08	0.003
BIOCARTA_CDC42RAC_PATHWAY	15	2.08	0.003
REACTOME_3_UTR_MEDIATED_TRANSLATIONAL_REGULATION	104	2.07	0.003
REACTOME_NONSENSE_MEDIATED_DECAY_ENHANCED_BY_THE_EXON_JUNCTION_COMPLEX	106	2.05	0.003
REACTOME_PEPTIDE_CHAIN_ELONGATION	85	2.03	0.004
REACTOME_ANTIVIRAL_MECHANISM_BY_IFN_STIMULATED_GENES	61	2.01	0.005
RIBONUCLEOPROTEIN_COMPLEX_BIOGENESIS_AND_ASSEMBLY	79	1.97	0.006
REACTOME_PROCESSING_OF_CAPPED_INTRON_CONTAINING_PRE_MRNA	132	1.97	0.006
REACTOME_INFLUENZA_VIRAL_RNA_TRANSCRIPTION_AND_REPLICATION	101	1.94	0.008
REACTOME_MRNA_PROCESSING	151	1.92	0.009
RNA_PROCESSING	159	1.88	0.013
BIOCARTA_G1_PATHWAY	25	1.84	0.017
RNA_SPLICING	87	1.82	0.020
REACTOME_MRNA_SPLICING	104	1.80	0.023
REACTOME_CELL_CYCLE	328	1.79	0.024
REACTOME_CELL_CYCLE_CHECKPOINTS	97	1.78	0.026
CELLULAR_COMPONENT_ASSEMBLY	272	1.75	0.030
REGULATION_OF_TRANSCRIPTION_FROM_RNA_POLYMERASE_II_PROMOTER	271	1.75	0.030
MRNA_METABOLIC_PROCESS	78	1.74	0.031
REACTOME_HIV_INFECTION	183	1.74	0.031
RANSCRIPTION_FROM_RNA_POLYMERASE_II_PROMOTER	427	1.73	0.032
REACTOME_SIGNALING_BY_BMP	21	1.72	0.032
REACTOME_TRANSCRIPTIONAL_ACTIVITY_OF_SMAD2_SMAD3_SMAD4_HETEROTRIMER	33	1.72	0.033
REACTOME_ACTIVATION_OF_THE_MRNA_UPON_BINDING_OF_THE_CAP_BINDING_COMPLEX_AND_EIFS_AND_SUBSEQUENT_BINDING_TO_43S	56	1.71	0.034
REACTOME_SIGNALING_BY_TGF_BETA_RECEPTOR_COMPLEX	56	1.70	0.037
REACTOME_CELL_CYCLE_MITOTIC	255	1.70	0.037
NUCLEAR_EXPORT	31	1.68	0.041
REACTOME_DOWNREGULATION_OF_SMAD2_3_SMAD4_TRANSCRIPTIONAL_ACTIVITY	18	1.68	0.041
POSITIVE_REGULATION_OF_NUCLEOBASENUCLEOSIDENUCLEOTIDE_AND_NUCLEIC_ACID_METABOLIC_PROCESS	143	1.68	0.042
KEGG_RNA_DEGRADATION	55	1.67	0.042
KEGG_ADHERENS_JUNCTION	70	1.67	0.043
G1_S_TRANSITION_OF_MITOTIC_CELL_CYCLE	25	1.67	0.045
REACTOME_TRANSPORT_OF_MATURE_TRANSCRIPT_TO_CYTOPLASM	51	1.66	0.045
POSITIVE_REGULATION_OF_TRANSCRIPTION	134	1.65	0.048
Downregulated			
None			

*ER* estrogen receptor, *NHS* Nurses’ Health Study, *NES* normalized enrichment score, *FDR* false discovery rate

^a^Only gene sets replicated in The Cancer Genome Atlas (TCGA) dataset and FDR <0.05 are shown

^b^Enriched gene sets for comparison of recent alcohol consumption 10+ vs. 0 g/day

In both ER+ and ER- tumors, alcohol intake was associated with upregulation in gene sets involved in RNA metabolism and transport and cell cycle. For instance, “REACTOME_METABOLISM_OF_RNA” was the top ranked pathway under the category of RNA metabolism and transport in both ER+ and ER- tumors (Fig. 3a and b). However, despite some overlapping gene sets within each category, there were some specific gene sets in either ER+ or ER- tumors. For example, among cell cycle related gene sets, in ER+ tumors, genes involved in the G2/M phase checkpoint were overexpressed while in ER- tumors, genes involved in the G1 or G1/S transition were upregulated (Fig. 3a and b). Further, as we hypothesized that the biological mechanism of the alcohol and breast cancer association may vary between ER+ and ER- tumors, we also noted that there were several ER+ or ER- tumor-specific gene sets. Among ER+ tumors, alcohol intake was linked to gene sets involved in upregulation of DNA repair but this not observed among ER- tumors. In addition, genes related to lipid metabolism were down-expressed in ER+ tumors but not in ER- tumors. On the other hand, upregulation in cytokine signaling was only observed among ER- tumors.

Among ER+ tumor-adjacent normal tissues, we observed significant enrichment for 335 pathway-defined gene sets (FDR <0.05), of which 1 was upregulated and 334 were downregulated by recent alcohol intake (i.e. 10+ vs. 0 g/day); among ER- tumor-adjacent normal tissues, 340 and 58 pathway-defined gene sets were significantly (FDR <0.05) upregulated and downregulated, respectively. Table 5 presents the top 10 ranked upregulated or downregulated pathway-defined gene sets identified in the NHS/NHSII. Among ER+ tumor-adjacent normal tissues, only one gene set (i.e. olfactory transduction) was significantly upregulated at FDR <0.05; the top ranked downregulated gene sets included mitochondrial respiratory electron transport and TCA cycle, WNT signaling pathway, integrin pathway and focal adhesion, and fatty acids/triacylglycerol/ketone body metabolism. Among ER- tumor-adjacent normal tissues, the top ranked upregulated gene sets, such as RNA metabolism and translation, were also seen among those top ranked in ER- tumors; the strongest enrichment for downregulated gene sets included neuroactive ligand-receptor interaction, GPCR ligand binding, and cytochrome P450 arranged by substrate type.

**Table 5:** Top 10 enriched gene sets^a^ by alcohol consumption^b^ in tumor-adjacent normal samples in the NHS and the NHSII

Pathway-defined gene set	Number of enriched genes	NES	FDR
ER+ tumor-adjacent normal tissues			
Upregulated			
KEGG_OLFACTORY_TRANSDUCTION	153	1.97	0.04
Downregulated			
REACTOME_TCA_CYCLE_AND_RESPIRATORY_ELECTRON_TRANSPORT	123	−2.33	<0.001
REACTOME_SIGNALING_BY_WNT	60	−2.33	<0.001
REACTOME_PYRUVATE_METABOLISM_AND_CITRIC_ACID_TCA_CYCLE	39	−2.30	<0.001
REACTOME_CTNNB1_PHOSPHORYLATION_CASCADE	15	−2.28	<0.001
KEGG_FOCAL_ADHESION	186	−2.27	<0.001
BIOCARTA_RHO_PATHWAY	30	−2.27	<0.001
BIOCARTA_INTEGRIN_PATHWAY	36	−2.26	<0.001
BIOCARTA_PYK2_PATHWAY	27	−2.26	<0.001
KEGG_PATHOGENIC_ESCHERICHIA_COLI_INFECTION	51	−2.25	<0.001
REACTOME_FATTY_ACID_TRIACYLGLYCEROL_AND_KETONE_BODY_METABOLISM	151	−2.24	1.55E-04
ER- tumor-adjacent normal			
Up-regulated			
REACTOME_METABOLISM_OF_MRNA	206	2.87	<0.001
REACTOME_METABOLISM_OF_RNA	248	2.79	<0.001
REACTOME_NONSENSE_MEDIATED_DECAY_ENHANCED_BY_THE_EXON_JUNCTION_COMPLEX	106	2.66	<0.001
REACTOME_INFLUENZA_LIFE_CYCLE	134	2.65	<0.001
REACTOME_3_UTR_MEDIATED_TRANSLATIONAL_REGULATION	104	2.64	<0.001
REACTOME_TRANSLATION	144	2.63	<0.001
REACTOME_SRP_DEPENDENT_COTRANSLATIONAL_PROTEIN_TARGETING_TO_MEMBRANE	108	2.62	<0.001
REACTOME_TRANSPORT_OF_MATURE_TRANSCRIPT_TO_CYTOPLASM	51	2.61	<0.001
REACTOME_PROCESSING_OF_CAPPED_INTRON_CONTAINING_PRE_MRNA	132	2.60	<0.001
REACTOME_MRNA_PROCESSING	151	2.59	<0.001
Down-regulated			
KEGG_NEUROACTIVE_LIGAND_RECEPTOR_INTERACTION	192	−2.52	<0.001
REACTOME_GPCR_LIGAND_BINDING	297	−2.49	<0.001
REACTOME_TRANSPORT_OF_GLUCOSE_AND_OTHER_SUGARS_BILE_SALTS_AND_ORGANIC_ACIDS_METAL_IONS_AND_AMINE_COMPOUNDS	76	−2.36	<0.001
REACTOME_CLASS_A1_RHODOPSIN_LIKE_RECEPTORS	212	−2.29	4.14E-04
REACTOME_POTASSIUM_CHANNELS	86	−2.30	5.18E-04
REACTOME_CYTOCHROME_P450_ARRANGED_BY_SUBSTRATE_TYPE	37	−2.20	2.04E-03
GENERATION_OF_A_SIGNAL_INVOLVED_IN_CELL_CELL_SIGNALING	27	−2.14	3.22E-03
REACTOME_VOLTAGE_GATED_POTASSIUM_CHANNELS	36	−2.14	3.29E-03
G_PROTEIN_SIGNALING_COUPLED_TO_CYCLIC_NUCLEOTIDE_SECOND_MESSENGER	83	−2.12	3.50E-03
REACTOME_G_ALPHA_I_SIGNALLING_EVENTS	147	−2.12	3.50E-03

*ER* estrogen receptor, *NHS* Nurses’ Health Study, *NES* normalized enrichment score, *FDR* false discovery rate

^a^Top 10 ranked gene sets at FDR <0.05 are shown for upregulation and downregulation, respectively

^b^Enriched gene sets for comparison of recent alcohol consumption 10+ vs. 0 g/day

As several enzymes, such as alcohol dehydrogenase (ADH) and aldehyde dehydrogenase (ALDH), are known to play an important role in alcohol metabolism, we specifically examined the expression of genes involved in alcohol metabolism in tumors and adjacent normal tissues. Most of these genes, including *ADH1B*, *ALDH1A1*, *ADH1C* and *ALDH2*, were significantly down-expressed in ER+ or ER- tumors compared to tumor-adjacent normal tissues (Table 6), although none showed significant differential expression by alcohol intake in either tissue type. For instance, among the seven alcohol metabolism genes included in our data, *ADH1B* showed the most reduced expression in ER+ or ER- tumors (fold change 0.40).

**Table 6:** Differential expression of alcohol metabolism genes in tumor and tumor-adjacent normal tissues in the NHS and the NHSII

			ER+ tumors vs. tumor-adjacent normal tissues			ER- tumors vs. tumor-adjacent normal tissues		
Probeset ID	Entrez ID	Symbol	Log2(FC)	*t* value^a^	FDR^b^	Log2(FC)	*t* value^a^	FDR^b^
TC0401141	125	*ADH1B*	−1.33	−21.1	2.92E-61	−1.32	−11.0	1.59E-14
TC0901044	216	*ALDH1A1*	−0.61	−16.7	2.77E-44	−0.58	−8.6	6.93E-11
TC0401142	126	*ADH1C*	−0.25	−14.6	3.80E-36	−0.29	−7.6	3.08E-09
TC1100272	847	*CAT*	−0.34	−10.2	1.99E-20	−0.32	−4.2	6.20E-04
TC1200702	217	*ALDH2*	−0.18	−7.0	6.53E-11	−0.20	−4.0	1.01E-03
TC0401137	128	*ADH5*	−0.11	−4.8	7.69E-06	−0.04	−0.7	6.00E-01
TC1000709	1571	*CYP2E1*	−0.02	−1.9	8.21E-02	−0.02	−0.6	7.02E-01

*ER* estrogen receptor, *NHS* Nurses’ Health Study, *FDR* false discovery rate, *FC* fold change

^a^The *t* values were obtained from paired *t* tests of tumor and adjacent normal tissues: 357 pairs of ER+ tumor and ER+ tumor-adjacent normal tissues, and 86 pairs for ER- tumor and ER- tumor-adjacent normal, respectively

^b^FDR was calculated across all the 25,979 probes

As more than half (i.e. 60%) of the tumors in the NHS/NHSII were stage I tumors while the majority of the tumors in TCGA were stage II or III, we further conducted stratified analyses according to tumor stage in secondary analyses. Specifically, we performed GSEA among stage II/III ER+ tumors in the NHS/NHSII and further validated in stage II/III ER+ tumors in TCGA; we were not able to conduct similar analysis among stage II/III ER- tumors because of the limited case numbers in TCGA. We found that the replicated enrichment signals in stage II/III ER+ tumors were very similar to those in all ER+ tumors in the NHS/NHSII (Additional file 2: Table S6).

## Discussion

To our knowledge, this is the first epidemiologic study to assess the association between pre-diagnostic alcohol consumption and breast tumor genome-wide gene expression. In the differential gene expression analysis by recent alcohol consumption, we did not find individual genes significantly upregulated or downregulated by alcohol after accounting for multiple comparisons. However, gene set analysis identified reproducible enriched pathway-defined gene sets in breast tumors. Specifically, recent alcohol intake of at least 10 g/day was linked to increased proliferation and lower lipid metabolism in ER+ tumors; among ER- tumors, in addition to an increase in proliferation, some further signals, including upregulation in cytokine signaling, such as interferon (IFN) and TGF-β signaling pathways, were noted.

Cohort studies generally support a stronger positive association among ER+ tumors than among ER- tumors [26, 27]. A strong enrichment signal observed from GSEA was increased proliferation in ER+ tumors. Several of the significantly upregulated gene sets, including cell cycle regulation (e.g. mitosis and G2/M checkpoint) and DNA repair are closely related to proliferation [28]. In addition, RNA processing (e.g. RNA splicing or transport) has been shown to affect cell cycle and proliferation [29], although increased RNA processing also may be a consequence of proliferation. Our finding is consistent with experimental studies in which ethanol promoted proliferation in ER+ breast tumor cell lines [30–32]. In ER+ tumors, we also observed downregulation of lipid metabolism, including the PPAR-gamma signaling pathway. PPAR-gamma signaling plays an essential role in adipocyte differentiation and expression of adipocyte specific genes, and also regulates lipid metabolism, cell proliferation and differentiation, glucose homeostasis and inflammation [33]. Further, in experimental studies, PPAR-gamma inhibited proliferation in ER+ breast cancer cell lines [34] and ethanol inhibited PPAR-gamma dependent transcriptional activation [35]. Taken together, downregulation of the PPAR-gamma signaling pathway is consistent with the observed increase in proliferation in our data. In addition, lower lipid metabolism was observed among ER+ tumor-adjacent normal tissues in the current dataset. If replicated, this finding suggests that alcohol consumption disrupts lipid metabolism, providing another possible link to alcohol-related breast pathogenesis.

Among the hypothesized mechanisms through which alcohol consumption increases breast cancer risk, particularly ER+ disease, the most studied pathway is estrogen metabolism with supporting evidence from intervention studies that alcohol drinking is associated with increases in circulating estrogens [5, 6]. In experimental studies, ER-mediated estrogen signaling can increase cell proliferation that in turn can induce genetic mutations [36, 37], while estrogen metabolites, independent of ER signaling, also can cause DNA damage [37]. Although our data are consistent with estrogen/ER signaling mediated increased proliferation and DNA damage, we did not find alcohol intake to be associated with increased expression of specific estrogen-related genes or gene sets in ER+ tumors or tumor-adjacent normal tissue. The reason is unclear. To what extent alcohol-associated estrogen metabolism occurs in breast tissue in cancer-free women and whether it would be preserved in breast tissue during tumor progression is not known. In a recent study that explored parity-associated gene expression signatures, the signature identified in normal breast tissue was preserved in ER+ but not in ER- breast tumors [13]. Further, as shown in alcohol intervention studies with a crossover study design [5, 6], the alcohol-associated increases in circulating estrogen or estrogen metabolite levels are a relatively acute alcohol effect. Whether alcohol-induced estrogen metabolism occurring in breast tissue is similarly short term or more long lasting is not known, and could have influenced our ability to detect an association.

Among ER- tumors, recent alcohol consumption was also linked to increases in proliferation. In addition to cell cycle upregulation, significant increases in translational and post-translational modification were observed, which may be associated with alterations in cell cycle and regulation of cell growth [38]. However, a prior experimental study reported that ethanol only induced proliferation in ER+ but not ER- breast cancer cell lines [31]. The observation of alcohol-related proliferation in both ER+ and ER- tumors in our data suggests that alcohol-induced proliferation may not exclusively act through estrogen metabolism, as no pathway-defined gene sets related to estrogen metabolism were significantly enriched by alcohol intake.

Compared to the enrichment signals observed among ER+ tumors, a distinct enrichment was found in ER- tumors: alcohol intake was associated with upregulation in cytokine signaling including IFN signaling and TGF-β signaling pathways. Alcohol is known to modulate the immune system in a complex way. In animal models, chronic ethanol exposure was shown to alter cytokine levels (e.g. TNF-α, TGF-β, IL-6) in a variety of tissues, including lung, liver and brain [39], although breast tissue was not assessed. In a population study of over 1300 women, circulating IL-6 levels significantly increased among women consuming at least one alcoholic drink per day, while no increase was reported among women with light alcohol intake (i.e. less than one drink/day) [40]. In addition, cytokines play an important role in breast tumor growth and progression [41, 42]. Expression levels of multiple cytokines were higher in ER- compared to ER+ breast tumors, including IFN-γ, TNF-α, and IL-6 and IL-8 [43]. Further, breast tumor ER expression may be an important mediator of the transition of TGF-β from tumor suppression to tumor promotion: loss of ER expression (i.e. in ER- tumor cells) and loss of hormonally controlled growth may lead to an increased tumor promoting effect of TGF-β [44]. Interestingly, the significantly enriched pathway-defined gene sets identified in ER- tumors had a consistent enrichment pattern and even stronger enrichment signals among ER- tumor-adjacent normal tissues, while no similarities were observed in enrichment signals between ER+ tumors and ER+ tumor-adjacent normal tissues. One possible reason is that ER- tumors in this dataset were on average more advanced than ER+ tumors (i.e. larger in size, higher in grade and at a more advanced stage); thus, although the tumor-adjacent normal tissues were defined as generally > 1 cm from the tumor edge, ER- tumors may have more strongly influenced adjacent normal tissues. However, the fact that adjacent normal tissues may contain information on the environment surrounding the tumors may not directly relate to alcohol consumption; thus, the exact reason for the consistent enrichment pattern in ER- tumors and adjacent normal tissues is not clear. As this is the first ever assessment of alcohol intake and gene expression in both tumor and adjacent normal tissues, replication of this finding in other studies will be important.

Although none of the known alcohol metabolism genes were differentially expressed by alcohol intake in breast tumors in this study, several of these genes, including *ADH1B*, *ADH1C*, *ALDH2* and *ALDH1A1* were substantially downregulated in tumors compared to tumor-adjacent normal tissues, regardless of tumor ER expression. Our results are consistent with a previous study that observed class I ADH (including *ADH1A*, *ADH1B* and *ADH1C*) to be more highly expressed, at both mRNA and protein levels, in normal breast tissues from cancer-free women than in invasive breast tumor tissues [45]. Despite no significant enrichment of pathway-defined gene sets involved in alcohol metabolism according to alcohol intake, it was interesting to see that recent alcohol consumption (i.e. 10+ g/day) was marginally associated with significant downregulation in retinol metabolism (i.e. KEGG_RETINOL_METABOLISM) among ER+ tumors (FDR = 0.11; also replicated in TCGA) because ADH enzymes are also involved in retinol metabolism [46]. Abnormal retinoid metabolism has been observed in several cancers, including breast cancer [47].

Our study, using tumor genome-wide gene expression profiling provided novel insights of alcohol-related molecular pathways in breast tumors. The evaluation was conducted within large prospective cohort studies with detailed data on alcohol consumption, covariates, and cancer diagnosis and tumor characteristics. In addition, this study has a sizable number of breast tumor and tumor-adjacent normal specimens. Further, our results, using microarrays, were validated in another platform using RNA-Seq. Finally, in addition to single-probe analysis, we conducted pathway analysis (i.e. GSEA), which has several advantages over single-gene analysis. For example, it makes interpretation easier by focusing on pathways and biological processes rather than single high-scoring genes which may be poorly annotated. GSEA also makes it possible to detect modest expression changes in individual genes as it can increase the signal-to-noise ratio. However, one drawback in this GSEA is that the *P* value estimation using gene permutation under GSEA “Preranked”’ function does not take into account correlation among genes [48].

Our study also has limitations. One limitation is the use of FFPE tissues for gene expression profiling, because FFPE can make retrieval of RNA challenging due to chemical modification of RNA and related RNA degradation. However, archived FFPE samples have been shown comparable to fresh-frozen samples in assessing differential expression in lung, colon and kidney tissues [49]. In addition, our results were validated in a dataset derived from fresh-frozen breast tumor samples that were part of TCGA. However, there were differences in patient characteristics (e.g. age) and tumor characteristics (i.e. tumor stage) between the NHS and TCGA datasets. Another limitation is the lack of a validation dataset for tumor-adjacent normal tissues. Further, women participating in this study had relatively low levels of alcohol consumption and thus we were not able to evaluate the effect of moderate to heavy alcohol intake on tumor gene expression. Indeed, in epidemiologic studies, compared to women without recent alcohol intake, those with recent consumption 10–20 g/day had only ~10% increased breast cancer risk while those consuming at least 30 g/day had > 30% increased risk [3]. Finally, the tumor gene expression profiling here may be a mixed profiling of malignant epithelial and stromal cells. Laser capture microdissection can be used to isolate specific cell types; however, it was not feasible considering the large sample size in this study.

## Conclusions

Our data suggest that alcohol consumption is associated with increased proliferation and lower lipid metabolism among ER+ breast tumors while among ER- breast tumors, alcohol consumption is not only linked to increased proliferation but also upregulation in cytokine signaling, particularly IFN and TGF-β signaling. Future studies of gene expression profiling in normal breast tissues from cancer-free women are of particular interest. Alcohol consumption is not considered a prognostic factor for breast cancer recurrence or death [50]. Assessment of the effect of alcohol consumption on normal breast tissues, together with profiling data from breast tumors and/or tumor-adjacent normal tissues will be critical in further elucidating the alcohol-related breast carcinogenesis. Furthermore, as alcohol is known to impact one-carbon metabolism and induce aberrant DNA methylation [51], integrating DNA methylation and gene expression data may provide deeper insights into the underlying biology of the association between alcohol and breast cancer.

## Additional files

Additional file 1: Figure S1. ESR1, PGR, ERBB2 mRNA expression by IHC ER, PR, and HER2, respectively, in the NHS and the NHSII. (PDF 273 kb)

Additional file 2: Table S1. Comparison of study population characteristics in the NHS/NHSII and TCGA. **Table S2.** Characteristics of invasive breast cancer cases by recent alcohol consumption in the TCGA. **Table S3.** Top 10 ranked differentially expressed probes by recent alcohol consumption in the NHS and the NHSII. **Table S4.** Enriched gene sets (FDR 0.05–0.1) by recent alcohol consumption in ER+ tumors in the NHS and the NHSII. **Table S5.** Enriched gene sets (FDR 0.05–0.1) by recent alcohol consumption in ER- tumors in the NHS and the NHSII. **Table S6.** Enriched gene sets by alcohol consumption in stage II/III ER+ tumors in the NHS and the NHSII. (DOCX 56 kb)

Additional file 3: Figure S2. Differentially expressed probes (N = 25,979) by alcohol consumption in the NHS and the NHSII. The top four figures show recent alcohol intake > 0 - < 10 vs. 0 g/day and the bottom four figures show recent alcohol intake 10+ vs. 0 g/day. (PDF 1418 kb)

## Abbreviations

ADH: Alcohol dehydrogenase
ALDH: Aldehyde dehydrogenase
AUC: Area under the curve
BMI: Body mass index
CGEMS: Cancer Genetic Markers of Susceptibility
CI: Confidence interval
ER: Estrogen receptor
FDR: False discovery rate
FFPE: Formalin-fixed paraffin-embedded
GO: Gene Ontology
GSEA: Gene set enrichment analysis
HER2: Human epidermal growth factor receptor 2
IFN: Interferon
IHC: Immunohistochemistry
IL: Interleukin
KEGG: Kyoto Encyclopedia of Genes and Genomes
LIMMA: Linear models for microarray data
MHT: Menopausal hormone therapy
MSigDB: Molecular Signatures Database
NES: Normalized enrichment score
NHS: Nurses’ Health Study
PR: Progesterone receptor
RMA: Robust multiarray average
RNA-Seq: RNA sequencing
RSEM: RNA-Seq by expectation maximization
TCGA: The Cancer Genome Atlas
TGF: Transforming growth factor
TNF: Tumor necrosis factor
