# Supplementary figures and images for: Alcohol consumption and breast tumor gene expression
Source: Breast Cancer Res. 2017 Sep 12;19:108. doi: 10.1186/s13058-017-0901-y (PMC5596493; doi:10.1186/s13058-017-0901-y)

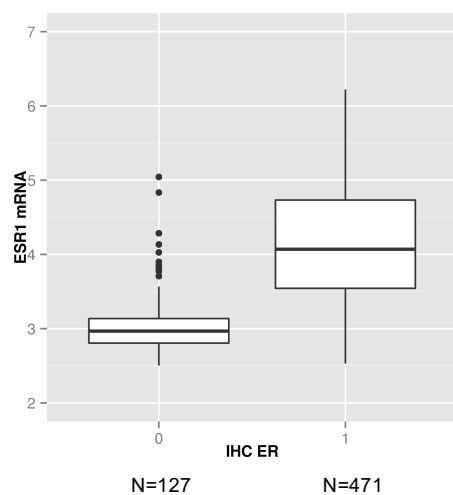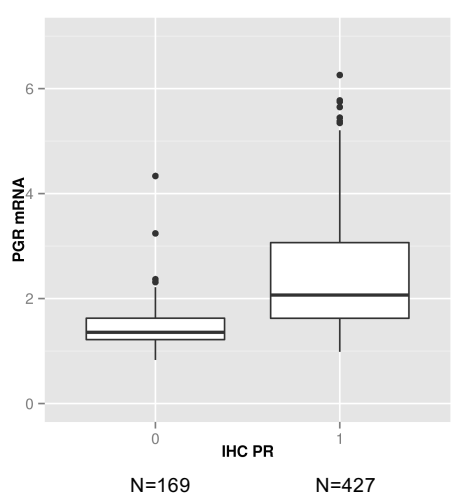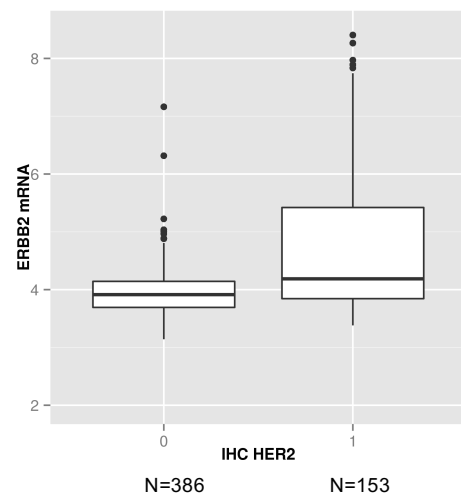

Supplement: Supplementary file 1 — ESR1, PGR, ERBB2 mRNA expression by IHC ER, PR, and HER2, respectively, in the NHS and the NHSII. (PDF 273 kb) [file 13058_2017_901_MOESM1_ESM.pdf]

ER+ tumors

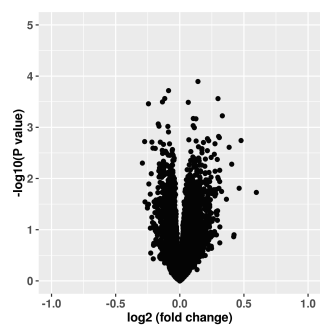

ER+ tumor adjacent normal

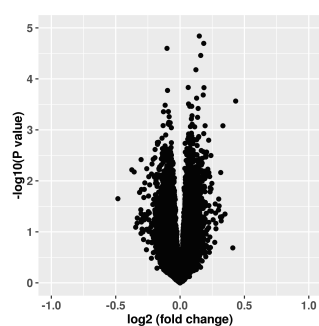

ER- tumors

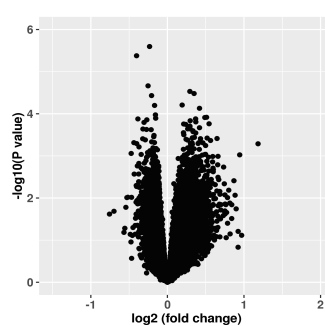

ER- tumor adjacent normal

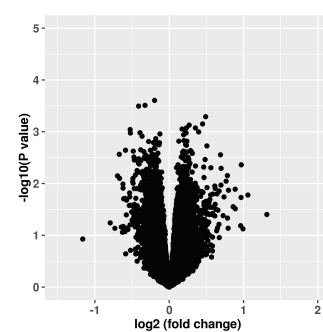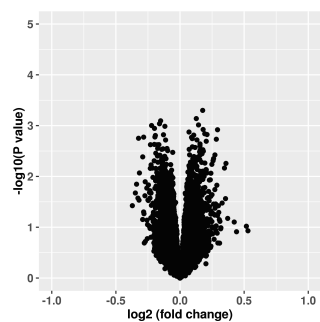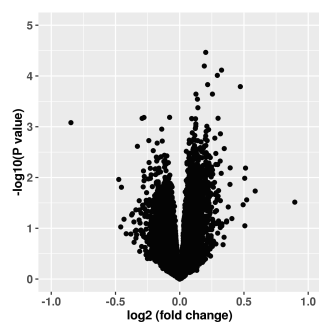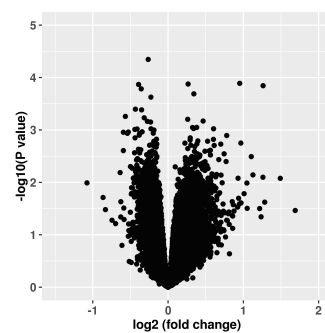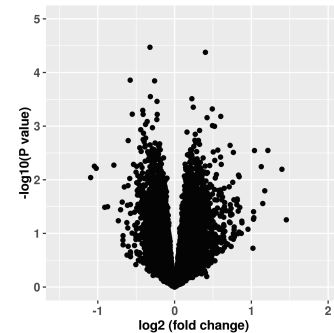

Supplement: Supplementary file 3 — Differentially expressed probes (N = 25,979) by alcohol consumption in the NHS and the NHSII. The top four figures show recent alcohol intake > 0 - < 10 vs. 0 g/day and the bottom four figures show recent alcohol intake 10+ vs. 0 g/day. (PDF 1418 kb) [file 13058_2017_901_MOESM3_ESM.pdf]
