# Supplementary material for: Alcohol consumption and breast tumor gene expression
Source: Breast Cancer Res. 2017 Sep 12;19:108. doi: 10.1186/s13058-017-0901-y (PMC5596493; doi:10.1186/s13058-017-0901-y)
Supplement: Supplementary file 2 — Comparison of study population characteristics in the NHS/NHSII and TCGA. Table S2. Characteristics of invasive breast cancer cases by recent alcohol consumption in the TCGA. Table S3. Top 10 ranked differentially expressed probes by recent alcohol consumption in the NHS and the NHSII. Table S4. Enriched gene sets (FDR 0.05–0.1) by recent alcohol consumption in ER+ tumors in the NHS and the NHSII. Table S5. Enriched gene sets (FDR 0.05–0.1) by recent alcohol consumption in ER- tumors in the NHS and the NHSII. Table S6. Enriched gene sets by alcohol consumption in stage II/III ER+ tumors in the NHS and the NHSII. (DOCX 56 kb) [file 13058_2017_901_MOESM2_ESM.docx]

**Supplementary Table 1. Comparison of study population characteristics in the NHS/NHSII and TCGA.**

|  | **NHS and NHSII**  **N=602** | |  | **TCGA**  **N=166** | |
| --- | --- | --- | --- | --- | --- |
|  | Mean | SD |  | Mean | SD |
| Age at diagnosis, y | 61 | 9.4 |  | 56 | 12.4 |
| BMI at diagnosis, kg/m^2^ | 26 | 5.0 |  | 28.7 | 6.9 |
| Parity | 2.6 | 1.8 |  | 2.6 | 1.2 |
| Recent alcohol, g/d | 6.4 | 11.4 |  | N/A^1^ | N/A^1^ |
|  |  |  |  |  |  |
| Recent alcohol, drink/day | N | % |  | N | % |
| 0 | 206 | 34.4 |  | 42 | 25.3 |
| <1 | 267 | 44.6 |  | 108 | 65.1 |
| 1+ | 126 | 21.0 |  | 16 | 9.6 |
| First degree family history | 96 | 16.0 |  | 32 | 19.3 |
| Menopausal at diagnosis |  |  |  |  |  |
| Pre-menopause | 103 | 17.2 |  | 63 | 38 |
| Post-menopause | 480 | 80.1 |  | 100 | 60.2 |
| Unknown | 16 | 2.7 |  | 3 | 1.8 |
| Current MHT use^2^ | 249 | 51.9 |  | 12 | 12.1 |
| Year of diagnosis |  |  |  |  |  |
| 1990-1999 | 345 | 57.6 |  | 0 | 0 |
| 2000-2004 | 208 | 34.7 |  | 15 | 9.0 |
| 2005-2009 | 46 | 7.7 |  | 133 | 80.1 |
| >2009 | 0 | 0 |  | 18 | 10.8 |
| ER |  |  |  |  |  |
| Positive | 471 | 78.8 |  | 133 | 80.1 |
| Negative | 127 | 21.2 |  | 33 | 19.9 |
| Stage |  |  |  |  |  |
| I | 362 | 60.6 |  | 37 | 22.3 |
| II | 181 | 30.3 |  | 101 | 60.8 |
| III | 49 | 8.2 |  | 28 | 16.9 |
| IV | 5 | 0.8 |  | 0 | 0 |

^1^N/A: not applicable.

^2^Among postmenopausal women.

**Supplementary table 2. Characteristics of invasive breast cancer cases by recent alcohol consumption, TCGA.**

|  | Recent alcohol consumption, drink/day | | | | | | | |
| --- | --- | --- | --- | --- | --- | --- | --- | --- |
|  | 0 | |  | < 1 | |  | 1+ | |
|  | N = 42 | |  | N = 108 | |  | N = 16 | |
|  | Mean | SD |  | Mean | SD |  | Mean | SD |
| Age at diagnosis, y | 58.5 | 13.2 |  | 55.0 | 12.1 |  | 56.3 | 12.7 |
| BMI at diagnosis, kg/m^2^ | 31.6 | 7.8 |  | 28.0 | 6.5 |  | 25.6 | 4.4 |
| Parity | 2.8 | 1.0 |  | 2.6 | 1.2 |  | 1.8 | 0.9 |
|  |  |  |  |  |  |  |  |  |
|  | N | % |  | N | % |  | N | % |
| First degree family history | 7 | 16.7 |  | 25 | 23.1 |  | 0 | 0 |
| Menopausal at diagnosis |  |  |  |  |  |  |  |  |
| Post-menopause | 29 | 68.3 |  | 63 | 61.5 |  | 8 | 50.0 |
| Pre-/Peri-menopause | 12 | 29.3 |  | 44 | 38.5 |  | 7 | 43.8 |
| Unknown | 1 | 2.4 |  | 1 | 0.9 |  | 1 | 6.2 |
| Current MHT use^1^ | 4 | 13.8 |  | 7 | 11.3 |  | 1 | 12.5 |
| Year of diagnosis |  |  |  |  |  |  |  |  |
| 2001-2007 | 20 | 47.6 |  | 48 | 44.4 |  | 6 | 37.5 |
| 2008-2011 | 22 | 52.4 |  | 60 | 55.6 |  | 10 | 62.5 |

^1^ Among postmenopausal women.

**Supplementary table 3. Top 10 ranked differentially expressed probes by recent alcohol consumption, NHS and NHSII.**

|  | **Recent alcohol consumption: >0 - <10 vs. 0 g/d** | | | | | |  |  | **Recent alcohol consumption:10+ vs. 0 g/d** | | | | | |  |
| --- | --- | --- | --- | --- | --- | --- | --- | --- | --- | --- | --- | --- | --- | --- | --- |
| Probeset_id | Entrez ID | Symbol | Log2(FC) | t value | P value | FDR |  | Probeset_id | Entrez ID | Symbol | Log2(FC) | t value | P value | FDR | |
| ***ER+ tumors*** |  |  |  |  |  |  |  |  |  |  |  |  |  |  | |
| TC1300067 | - | - | 0.14 | 3.87 | 1.27E-04 | 1.00 |  | TC0X01310 | 23641 | LDOC1 | 0.18 | 3.51 | 5.02E-04 | 1.00 | |
| TC1201526 | 9891 | NUAK1 | -0.09 | -3.76 | 1.92E-04 | 1.00 |  | TC0201752 | 55654 | TMEM127 | 0.13 | 3.40 | 7.27E-04 | 1.00 | |
| TC0600459 | - | - | -0.12 | -3.67 | 2.74E-04 | 1.00 |  | TC0300578 | - | - | -0.15 | -3.37 | 8.05E-04 | 1.00 | |
| TC1701111 | - | - | 0.30 | 3.67 | 2.75E-04 | 1.00 |  | TC1500529 | - | - | -0.16 | -3.34 | 9.18E-04 | 1.00 | |
| TC1500604 | - | - | -0.14 | -3.63 | 3.19E-04 | 1.00 |  | TC1500567 | - | - | 0.14 | 3.32 | 9.70E-04 | 1.00 | |
| TC0X01336 | 3423 | IDS | 0.07 | 3.62 | 3.25E-04 | 1.00 |  | TC1200555 | - | - | -0.22 | -3.31 | 9.96E-04 | 1.00 | |
| TC0900273 | - | - | -0.24 | -3.61 | 3.47E-04 | 1.00 |  | TC0801051 | 23462 | HEY1 | -0.12 | -3.30 | 1.03E-03 | 1.00 | |
| TC1101542 | - | - | 0.33 | 3.46 | 5.97E-04 | 1.00 |  | TC0201249 | - | - | -0.20 | -3.28 | 1.13E-03 | 1.00 | |
| TC1300271 | 84945 | ABHD13 | 0.11 | 3.42 | 6.73E-04 | 1.00 |  | TC0801210 | 4982 | TNFRSF11B | 0.18 | 3.26 | 1.19E-03 | 1.00 | |
| TC0201319 | - | - | 0.13 | 3.42 | 6.83E-04 | 1.00 |  | TC1000078 | - | - | 0.29 | 3.26 | 1.21E-03 | 1.00 | |
|  |  |  |  |  |  |  |  |  |  |  |  |  |  |  | |
| ***ER+ tumor adjacent normal*** | | |  |  |  |  |  |  |  |  |  |  |  |  | |
| TC0301864 | 1370 | CPN2 | 0.15 | 4.39 | 1.45E-05 | 0.22 |  | TC0X01283 | 541465 | CT45A6 | 0.20 | 4.19 | 3.43E-05 | 0.63 | |
| TC1100038 | - | - | 0.18 | 4.32 | 2.01E-05 | 0.22 |  | TC1700314 | - | - | 0.19 | 4.04 | 6.37E-05 | 0.63 | |
| TC0102536 | 128338 | DRAM2 | -0.10 | -4.27 | 2.52E-05 | 0.22 |  | TC0500049 | - | - | 0.33 | 4.00 | 7.67E-05 | 0.63 | |
| TC0102769 | 353145 | LCE3E | 0.16 | 4.19 | 3.47E-05 | 0.23 |  | TC1701511 | - | - | 0.29 | 3.94 | 9.74E-05 | 0.63 | |
| TC1701673 | 10871 | CD300C | 0.12 | 4.03 | 6.63E-05 | 0.34 |  | TC1901115 | - | - | 0.22 | 3.83 | 1.48E-04 | 0.70 | |
| TC0301003 | 401052 | LOC401052 | 0.06 | 3.83 | 1.47E-04 | 0.55 |  | TC0800027 | - | - | 0.47 | 3.81 | 1.62E-04 | 0.70 | |
| TC0501265 | - | - | 0.19 | 3.83 | 1.49E-04 | 0.55 |  | TC1500628 | - | - | 0.25 | 3.72 | 2.27E-04 | 0.74 | |
| TC0102227 | 64756 | ATPAF1 | -0.10 | -3.80 | 1.69E-04 | 0.55 |  | TC1701177 | 147011 | PROCA1 | 0.13 | 3.72 | 2.28E-04 | 0.74 | |
| TC0901462 | - | - | 0.18 | 3.75 | 2.07E-04 | 0.60 |  | TC2100374 | - | - | 0.14 | 3.66 | 2.87E-04 | 0.83 | |
| TC1700508 | 2535 | FZD2 | 0.13 | 3.71 | 2.39E-04 | 0.60 |  | TC0201091 | 151306 | GPBAR1 | 0.14 | 3.56 | 4.22E-04 | 0.99 | |
|  |  |  |  |  |  |  |  |  |  |  |  |  |  |  | |
| ***ER- tumors*** |  |  |  |  |  |  |  |  |  |  |  |  |  |  | |
| TC0601200 | 6954 | TCP11 | -0.23 | -4.98 | 2.53E-06 | 0.05 |  | TC0X00727 | 8225 | GTPBP6 | -0.26 | -4.26 | 4.51E-05 | 0.71 | |
| TC0600314 | - | - | -0.40 | -4.86 | 4.20E-06 | 0.05 |  | TC1501129 | - | - | 0.95 | 3.98 | 1.29E-04 | 0.71 | |
| TC1400632 | 122616 | C14orf79 | -0.25 | -4.45 | 2.17E-05 | 0.16 |  | TC1900198 | 126068 | ZNF441 | 0.27 | 3.97 | 1.33E-04 | 0.71 | |
| TC0200578 | 10461 | MERTK | 0.29 | 4.37 | 2.95E-05 | 0.16 |  | TC2000262 | - | - | -0.39 | -3.96 | 1.36E-04 | 0.71 | |
| TC0401447 | 3660 | IRF2 | 0.35 | 4.34 | 3.31E-05 | 0.16 |  | TC0901132 | 54829 | ASPN | 1.26 | 3.95 | 1.43E-04 | 0.71 | |
| TC0X00677 | 7739 | ZNF185 | -0.21 | -4.31 | 3.70E-05 | 0.16 |  | TC0103187 | 79098 | C1orf116 | -0.36 | -3.91 | 1.64E-04 | 0.71 | |
| TC0100103 | 116362 | RBP7 | 0.19 | 4.17 | 6.20E-05 | 0.21 |  | TC0601329 | 9697 | TRAM2 | 0.34 | 3.85 | 2.05E-04 | 0.76 | |
| TC0301301 | 200844 | C3orf67 | -0.17 | -4.17 | 6.34E-05 | 0.21 |  | TC0X00677 | 7739 | ZNF185 | -0.23 | -3.81 | 2.36E-04 | 0.77 | |
| TC0501344 | 5201 | PFDN1 | 0.42 | 4.13 | 7.41E-05 | 0.21 |  | TC0501449 | 153745 | FAM71B | -0.43 | -3.66 | 4.01E-04 | 0.81 | |
| TC1000695 | 80313 | LRRC27 | -0.16 | -4.03 | 1.06E-04 | 0.21 |  | TC1500717 | - | - | -0.35 | -3.65 | 4.14E-04 | 0.81 | |
|  |  |  |  |  |  |  |  |  |  |  |  |  |  |  | |
| ***ER- tumor adjacent normal*** | | |  |  |  |  |  |  |  |  |  |  |  |  | |
| TC0X00315 | - | - | -0.20 | -3.84 | 2.48E-04 | 0.95 |  | TC1400500 | 283600 | SLC25A47 | -0.32 | -4.40 | 3.38E-05 | 0.55 | |
| TC0400236 | 79644 | SRD5A3 | -0.33 | -3.78 | 3.09E-04 | 0.95 |  | TC0X00120 | 652904 | CKS1BP6 | 0.40 | 4.34 | 4.20E-05 | 0.55 | |
| TC1300620 | - | - | -0.41 | -3.77 | 3.20E-04 | 0.95 |  | TC1701542 | - | - | -0.58 | -4.01 | 1.39E-04 | 0.88 | |
| TC1400368 | - | - | 0.49 | 3.63 | 5.12E-04 | 0.95 |  | TC1400469 | - | - | -0.26 | -4.00 | 1.43E-04 | 0.88 | |
| TC1200246 | - | - | 0.45 | 3.53 | 7.07E-04 | 0.95 |  | TC1700502 | 92591 | ASB16 | -0.32 | -3.80 | 2.82E-04 | 0.88 | |
| TC0701752 | 474344 | GIMAP6 | 0.27 | 3.51 | 7.45E-04 | 0.95 |  | TC1800138 | 10982 | MAPRE2 | 0.22 | 3.78 | 3.08E-04 | 0.88 | |
| TC1600618 | 8139 | GAN | 0.23 | 3.47 | 8.43E-04 | 0.95 |  | TC0X00315 | - | - | -0.23 | -3.74 | 3.46E-04 | 0.88 | |
| TC0201590 | 27247 | NFU1 | 0.35 | 3.47 | 8.46E-04 | 0.95 |  | TC0400507 | 5393 | EXOSC9 | 0.24 | 3.67 | 4.44E-04 | 0.88 | |
| TC1800138 | 10982 | MAPRE2 | 0.17 | 3.46 | 8.88E-04 | 0.95 |  | TC2200755 | - | - | 0.49 | 3.65 | 4.78E-04 | 0.88 | |
| TC1300460 | - | - | -0.53 | -3.45 | 9.07E-04 | 0.95 |  | TC1600428 | - | - | -0.41 | -3.63 | 5.09E-04 | 0.88 | |

**Supplementary table 4. Enriched gene sets (FDR 0.05-0.1)^1^ by recent alcohol consumption^2^ in ER+ tumors, NHS and NHSII.**

| **Pathway-defined gene set** | **No. of enriched genes** | **NES^3^** | **FDR** |
| --- | --- | --- | --- |
| RIBONUCLEOPROTEIN_COMPLEX_BIOGENESIS_AND_ASSEMBLY | 79 | 1.76 | 0.051 |
| KEGG_DNA_REPLICATION | 29 | 1.77 | 0.051 |
| REACTOME_MRNA_PROCESSING | 151 | 1.76 | 0.052 |
| MRNA_METABOLIC_PROCESS | 78 | 1.75 | 0.054 |
| DNA_REPAIR | 109 | 1.75 | 0.055 |
| REACTOME_G2_M_CHECKPOINTS | 30 | 1.74 | 0.061 |
| REACTOME_CHROMOSOME_MAINTENANCE | 89 | 1.73 | 0.068 |
| REACTOME_DNA_STRAND_ELONGATION | 24 | 1.69 | 0.077 |
| RESPONSE_TO_DNA_DAMAGE_STIMULUS | 141 | 1.65 | 0.097 |
| REACTOME_NCAM1_INTERACTIONS | 38 | -1.90 | 0.058 |

^1^Only replicated gene sets at FDR 0.05-0.1 are presented here and those replicated gene sets at FDR<0.05 are presented in Table 3.

^2^Enriched gene sets for comparison of recent alcohol consumption 10+ g/d vs. 0.

^3^NES: normalized enrichment score.

**Supplementary table 5. Enriched gene sets (FDR 0.05-0.1)^1^ by recent alcohol consumption^2^ in ER- tumors, NHS and NHSII.**

| **Pathway-defined gene set** | **No. of enriched genes** | **NES^3^** | **FDR** |
| --- | --- | --- | --- |
| REACTOME_DEADENYLATION_OF_MRNA | 17 | 1.63 | 0.052 |
| ORGANELLE_ORGANIZATION_AND_BIOGENESIS | 427 | 1.63 | 0.052 |
| REACTOME_AMINO_ACID_SYNTHESIS_AND_INTERCONVERSION_TRANSAMINATION | 15 | 1.63 | 0.053 |
| REACTOME_MITOTIC_M_M_G1_PHASES | 133 | 1.62 | 0.056 |
| NUCLEOCYTOPLASMIC_TRANSPORT | 84 | 1.62 | 0.056 |
| MRNA_PROCESSING_GO_0006397 | 67 | 1.60 | 0.059 |
| NUCLEAR_TRANSPORT | 85 | 1.60 | 0.061 |
| REGULATION_OF_TRANSCRIPTIONDNA_DEPENDENT | 426 | 1.59 | 0.062 |
| BIOCARTA_ATRBRCA_PATHWAY | 17 | 1.59 | 0.062 |
| REACTOME_NEGATIVE_REGULATORS_OF_RIG_I_MDA5_SIGNALING | 26 | 1.59 | 0.062 |
| REACTOME_FORMATION_OF_THE_TERNARY_COMPLEX_AND_SUBSEQUENTLY_THE_43S_COMPLEX | 49 | 1.58 | 0.063 |
| REACTOME_TRANSPORT_OF_MATURE_MRNA_DERIVED_FROM_AN_INTRONLESS_TRANSCRIPT | 31 | 1.59 | 0.063 |
| REGULATION_OF_RNA_METABOLIC_PROCESS | 434 | 1.58 | 0.066 |
| REACTOME_TRANSPORT_OF_RIBONUCLEOPROTEINS_INTO_THE_HOST_NUCLEUS | 26 | 1.56 | 0.069 |
| REACTOME_NEP_NS2_INTERACTS_WITH_THE_CELLULAR_EXPORT_MACHINERY | 26 | 1.55 | 0.074 |
| POSITIVE_REGULATION_OF_RNA_METABOLIC_PROCESS | 113 | 1.54 | 0.077 |
| REACTOME_CHROMOSOME_MAINTENANCE | 89 | 1.53 | 0.080 |
| REACTOME_INTERACTIONS_OF_VPR_WITH_HOST_CELLULAR_PROTEINS | 31 | 1.51 | 0.087 |
| RNA_SPLICINGVIA_TRANSESTERIFICATION_REACTIONS | 32 | 1.49 | 0.092 |
| REACTOME_DNA_REPLICATION | 149 | 1.49 | 0.092 |
| KEGG_CELL_CYCLE | 106 | 1.49 | 0.092 |
| REGULATION_OF_DNA_METABOLIC_PROCESS | 39 | 1.49 | 0.093 |
| DNA_METABOLIC_PROCESS | 220 | 1.49 | 0.093 |
| REACTOME_MITOTIC_PROMETAPHASE | 64 | 1.48 | 0.096 |

^1^Only replicated gene sets at FDR 0.05-0.1 are presented here and those replicated gene sets at FDR<0.05 are presented in Table 4.

^2^Enriched gene sets for comparison of recent alcohol consumption 10+ g/d vs. 0.

^3^NES: normalized enrichment score.

**Supplementary table 6. Enriched gene sets^1^ by alcohol consumption^2^ in stage II/III ER+ tumors, NHS and NHSII.**

| **Pathway-defined gene set** | **No. of enriched genes** | **NES^3^** | **FDR** |
| --- | --- | --- | --- |
| **Up-regulated** |  |  |  |
| REACTOME_DOUBLE_STRAND_BREAK_REPAIR | 18 | 2.14 | 0.0005 |
| REACTOME_TRANSCRIPTION | 177 | 2.08 | 0.001 |
| REACTOME_MEIOSIS | 82 | 2.01 | 0.002 |
| REACTOME_DEPOSITION_OF_NEW_CENPA_CONTAINING_NUCLEOSOMES_AT_THE_CENTROMERE | 44 | 2.00 | 0.002 |
| REACTOME_MEIOTIC_RECOMBINATION | 58 | 1.99 | 0.002 |
| REACTOME_CHROMOSOME_MAINTENANCE | 89 | 1.97 | 0.002 |
| REACTOME_RNA_POL_I_RNA_POL_III_AND_MITOCHONDRIAL_TRANSCRIPTION | 97 | 1.95 | 0.003 |
| REGULATION_OF_TRANSCRIPTION_FROM_RNA_POLYMERASE_II_PROMOTER | 271 | 1.94 | 0.003 |
| MEIOTIC_CELL_CYCLE | 24 | 1.93 | 0.003 |
| POSITIVE_REGULATION_OF_RNA_METABOLIC_PROCESS | 113 | 1.88 | 0.005 |
| TRANSCRIPTION_INITIATION | 34 | 1.85 | 0.007 |
| REACTOME_RNA_POL_I_TRANSCRIPTION | 66 | 1.84 | 0.007 |
| POSITIVE_REGULATION_OF_TRANSCRIPTIONDNA_DEPENDENT | 111 | 1.84 | 0.007 |
| STEROID_HORMONE_RECEPTOR_SIGNALING_PATHWAY | 18 | 1.82 | 0.008 |
| REACTOME_MEIOTIC_SYNAPSIS | 56 | 1.82 | 0.008 |
| TRANSCRIPTION_INITIATION_FROM_RNA_POLYMERASE_II_PROMOTER | 28 | 1.80 | 0.009 |
| POSITIVE_REGULATION_OF_TRANSCRIPTION_FROM_RNA_POLYMERASE_II_PROMOTER | 61 | 1.79 | 0.009 |
| REACTOME_TELOMERE_MAINTENANCE | 58 | 1.79 | 0.009 |
| POSITIVE_REGULATION_OF_TRANSCRIPTION | 134 | 1.79 | 0.010 |
| INTRACELLULAR_RECEPTOR_MEDIATED_SIGNALING_PATHWAY | 19 | 1.78 | 0.010 |
| REACTOME_RNA_POL_I_PROMOTER_OPENING | 43 | 1.75 | 0.014 |
| REACTOME_PACKAGING_OF_TELOMERE_ENDS | 37 | 1.71 | 0.017 |
| REACTOME_MRNA_3_END_PROCESSING | 33 | 1.68 | 0.021 |
| REACTOME_RORA_ACTIVATES_CIRCADIAN_EXPRESSION | 23 | 1.65 | 0.028 |
| RNA_EXPORT_FROM_NUCLEUS | 18 | 1.64 | 0.029 |
| REACTOME_RNA_POL_I_TRANSCRIPTION_INITIATION | 22 | 1.60 | 0.037 |
| KEGG_NUCLEOTIDE_EXCISION_REPAIR | 38 | 1.58 | 0.042 |
| REACTOME_CIRCADIAN_REPRESSION_OF_EXPRESSION_BY_REV_ERBA | 21 | 1.53 | 0.056 |
| REACTOME_SIGNALING_BY_HIPPO | 20 | 1.51 | 0.063 |
| BIOCARTA_TGFB_PATHWAY | 18 | 1.44 | 0.094 |
| **Down-regulated** |  |  |  |
| KEGG_RETINOL_METABOLISM | 26 | -2.37 | 0.0004 |
| REACTOME_LIPID_DIGESTION_MOBILIZATION_AND_TRANSPORT | 38 | -2.32 | 0.001 |
| KEGG_PPAR_SIGNALING_PATHWAY | 58 | -2.22 | 0.004 |
| REACTOME_PHASE1_FUNCTIONALIZATION_OF_COMPOUNDS | 50 | -2.15 | 0.006 |
| REACTOME_CYTOCHROME_P450_ARRANGED_BY_SUBSTRATE_TYPE | 37 | -2.05 | 0.015 |
| EXCRETION | 29 | -1.98 | 0.020 |
| REACTOME_LIPOPROTEIN_METABOLISM | 23 | -1.83 | 0.044 |
| REACTOME_GPCR_LIGAND_BINDING | 297 | -1.79 | 0.054 |
| HORMONE_METABOLIC_PROCESS | 21 | -1.78 | 0.054 |
| REACTOME_GPCR_DOWNSTREAM_SIGNALING | 480 | -1.77 | 0.056 |
| REACTOME_CLASS_A1_RHODOPSIN_LIKE_RECEPTORS | 212 | -1.76 | 0.059 |
| REACTOME_ACYL_CHAIN_REMODELLING_OF_PC | 20 | -1.73 | 0.072 |
| REACTOME_BIOLOGICAL_OXIDATIONS | 91 | -1.71 | 0.074 |
| CATION_TRANSPORT | 114 | -1.69 | 0.080 |

^1^Only replicated gene sets at FDR < 0.1 are presented.

^2^Enriched gene sets for comparison of recent alcohol consumption 10+ g/d vs. 0.

^3^NES: normalized enrichment score.
